# Developmental changes of fluconazole clearance in neonates and infants in relation to ontogeny of glomerular filtration rate: literature review and data analysis

**DOI:** 10.1186/s40780-018-0103-5

**Published:** 2018-03-20

**Authors:** Kazutoshi Murakoso, Ryoichi Minagawa, Hirotoshi Echizen

**Affiliations:** 1 Department of Pharmacotherapy, Meiji Pharmaceutical University, 2-522-1 Noshio, Kiyose, Tokyo 204-8588 Japan; 2 Department of Hospital Pharmacy, Soka Municipal Hospital, 2-21-1 Soka, Saitama, 340-8560 Japan

**Keywords:** Neonates, Infants, Postmenstrual age, Fluconazole, Pharmacokinetics, Ontogeny, Glomerular filtration rate

## Abstract

**Background:**

Fluconazole is frequently prescribed for the treatment of systemic fungal infection in neonates and infants. At present, prediction of fluconazole doses according to developmental changes in fluconazole clearance is not being done in these patients. We aimed to formulate a developmental model of fluconazole clearance taking into account the ontogeny of renal function, since the drug is largely eliminated renally.

**Methods:**

We systematically retrieved the data of fluconazole pharmacokinetics and renal function in children and adults from databases (MEDLINE and Japan Medical Abstracts Society). Datasets were retrieved from individual children or groups from 9 studies comprising 55 neonates or infants at postmenstrual age (PMA) 27–58 weeks. Datasets were retrieved from 5 studies comprising 60 children and from 13 studies comprising 152 adults. Datasets of glomerular filtration rate (GFR) for individual pediatric subjects were retrieved from 4 studies comprising 187 neonates or infants.

**Results:**

Fluconazole clearance normalized to body surface area (BSA) (CL_BSA_) in neonates was 1/3 to 1/4 of adult values, but CL_BSA_ increased rapidly during the neonatal and infantile periods and attained near adult values at PMA 60 weeks. A significant correlation between CL_BSA_ and PMA was observed in neonates and infants: CL_BSA_ (mL/min/m^2^) = 0.26・ PMA (weeks) – 4.9 (*r* = 0.68, *p* < 0.001). In addition, the developmental time course of GFR normalized to BSA (GFR_BSA_) was fitted well to a sigmoidal model with the maximum GFR_BSA_ of 149 mL/min/1.73m^2^, PMA associated with 50% of GFR_BSA,max_ (PMA_50_) of 54 weeks, and the Hill coefficient of 3.7. A significant correlation between fluconazole clearance and GFR was found in neonates and infants: CL (mL/min) = 0.34・GFR (mL/min) – 0.53 (*r* = 0.84, *p* < 0.001). Assuming that plasma drug concentrations required for treating fungal infection are comparable between children and adults, fluconazole doses for pediatric patients with given PMAs may be predicted from adult doses (such as 100 mg/day) using size-normalized clearance as a scaling factor. The predicted doses for neonates and infants were largely within the ranges recommended in the prescribing information.

**Conclusions:**

The present study indicates that fluconazole doses for neonates and infants may be predicted from developmental change of systemic clearance, the ontogeny of which parallels the maturation of nephron function.

## Background

Numerous studies have been conducted to establish a comprehensive dosing formula for neonates, infants, and children who undergo tremendous developmental changes in pharmacokinetics and pharmacodynamics [1]. Recently, Anderson and Holford [2] reported that adult doses may be scaled down to pediatric doses using a child/adult body surface area (BSA) ratio or an allometric scaling factor (exponent of 3/4 of the child/adult body weight ratio) for children older than 2 or 3 years, but additional factor of maturation should also be taken into account for neonates and infants. However, whether the above approaches work well in predicting fluconazole doses for neonates and infants remains largely unknown.

Fluconazole is a triazole antifungal agent frequently used for the treatment of systemic candidiasis or cryptococcosis in pediatric patients including neonates and infants, at doses of 3 to 6 mg/kg [3]. However, these dosing schemes are largely empirical and are not supported by pharmacokinetic data. At present, no dosing formula has been established for estimating fluconazole doses considering developmental changes of systemic clearance (CL) of fluconazole.

With this background, we aimed to study the developmental time courses of fluconazole CL and glomerular filtration rate (GFR) as a function of postmenstrual age (PMA) in pediatric patients, using datasets retrieved systematically from relevant patient populations and adults using databases in English (MEDLINE) and Japanese (Japan Medical Abstract Society). Furthermore, we aimed to formulate an equation to estimate fluconazole doses for pediatric patients as a function of PMA by combining the relationship between CL and GFR and that between GFR and PMA in children. Here, we present the data indicating that fluconazole doses for neonates and infants may be predicted by our formula by comparing with the doses predicted using empirical formulas of Augsberger [4] and Crawford [5] as well as those recommended in the current prescribing information [3].

## Methods

### Search for pharmacokinetic data of fluconazole

We searched for studies on fluconazole pharmacokinetics performed in premature and full-term neonates, infants, children, and adults in the MEDLINE database using at a combination of the following keywords: “UK49858 OR fluconazole” AND “child* OR pediatric* OR infant* OR neonate* OR premature*” AND “pharmacokinetic*”. Additionally, we searched for relevant studies performed in adults using keywords of “UK49858 OR fluconazole” AND adult* AND pharmacokinetic*. We also searched for relevant studies reported in Japanese in the database operated by Japan Medical Abstracts Society, using the same set of keywords in Japanese. We also searched for the articles cited in the retrieved literature. We collected CL of fluconazole in studies in which the drug was administered intravenously. In articles that did not report CL but included data of area under the curve from time 0 to infinity (AUC_0-inf_) after intravenous injection, we calculated CL by dividing AUC_0-inf_ by the corresponding doses. When plasma concentration–time data were available after oral administration, systemic clearance was estimated by assuming that oral bioavailability of the drug was 94%, as reported elsewhere [6]. When only AUC from time 0 to 24 h (AUC_0–24_) were available, AUC were estimated by adding AUC_24-∞_ estimated by extrapolation from concentration measured at 24 h and the elimination constant.

### Developmental changes of fluconazole clearance and GFR as a function of PMA

Fluconazole CL in pediatric patients retrieved from the literature were size-corrected or normalized to body weight (BW) and body surface area (BSA) (CL_BW_ and CL_BSA_, respectively) as scaling factors for each patient. In the literature, individual body weights were available for most of the full-term neonates, but heights were available from approximately 30% of them. For non-Japanese neonates or infants at PMA 40 weeks or older without individual height data, the 50 percentile height for the corresponding chronological age according to the WHO growth chart for boys was used [7]. For Japanese infants, the growth chart for Japanese boys was used [8]. For most premature neonates, however, individual body weights and heights were not available in the literature. Their body weights and heights were estimated using Ahn’s data table that lists the average values based on the records of 5014 premature infants [9], irrespective of ethnicity.

BSA for full-term infants was estimated according to the formula of Haycock et al. [10] as follows:

1 $$ \mathrm{BSA}\ \left({\mathrm{m}}^2\right)=\mathrm{weight}\ {\left(\mathrm{kg}\right)}^{0.5378}\times \mathrm{height}\ {\left(\mathrm{cm}\right)}^{0.3964}\times 0.024265 $$

For children and adults whose individual body weights and heights were available, BSA was estimated according to the formula of DuBois and DuBois [11].

2 $$ \mathrm{BSA}\ \left({\mathrm{m}}^2\right)=\mathrm{weight}\ {\left(\mathrm{kg}\right)}^{0.425}\times \mathrm{height}\ {\left(\mathrm{cm}\right)}^{0.725}\times 0.07184 $$

In 13 articles on adult patients, the data of heights and weights were available in three articles comprising 39 individual patients. BSA of the respective patients were calculated using Eq. 2. In four articles, only mean heights and weights for the study populations were available. In these cases, group BSA was calculated using Eq. 2. In the remaining 6 articles, no height and weight data were available. In these cases, we assumed that the patients had standard body weight (63 kg) and height (170 cm), and hence BSA of 1.73 m^2^.

Developmental changes of fluconazole CL in premature and full-term neonates at PMA 60 weeks or younger were analyzed by a linear regression model as a function of PMA. For children aged above 5 years and adults, a linear regression model was used for the analysis using chronological age.

### Developmental model for GFR

We searched for articles that reported individual GFR and demographic data (body weight and height) of neonates or infants. While individual body weights were available for all subjects, heights were unavailable in 3 of 4 articles. Therefore, for the analysis on the development of GFR, we estimated BSA according to the Neo-BSA_w_ formula reported by Ahn [9].

3 $$ \mathrm{BSA}\ \left({\mathrm{cm}}^2\right)=10.602\times \mathrm{Weight}\ {\left(\mathrm{g}\right)}^{0.6561} $$

Retrieved GFR (unadjusted for size) and GFR normalized to BSA (GFR_BSA_) were fitted to a sigmoidal hyperbolic model as a function of PMA using the non-linear least squares regression method.

4 $$ {GFR}_{(t)}=\frac{GFR_{max}\cdot {\mathrm{PMA}}^n}{PMA^n+{PMA_{50}}^n} $$

where GFR_(t)_ is GFR at a given PMA, GFR_max_ is the maximum GFR, PMA_50_ is the PMA associated with 50% of GFR_max_, and n is the Hill coefficient indicating the steepness of the curve. We used this equation to estimate GFR of pediatric patients with fluconazole CL retrieved from the literature, since a previous study [12] showed that the developmental changes of GFR as a function of PMA were fitted well to the sigmoidal model.

### Comparisons of fluconazole doses for neonates and infants estimated by empirical formulas with those predicted by the present CL-based formulas and those in prescribing information

We assumed that fluconazole CL in a representative adult is 0.23 mL/min/kg [13] and the corresponding standard daily dose of the drug is 100 mg daily for most systemic fungal infections [3]. Also assuming that average plasma concentrations required for eliminating susceptible pathogens are largely similar between adults and children, we estimated fluconazole doses for neonates and infants with PMA 28–60 weeks by multiplying the standard adult dose (100 mg/day) by a ratio of fluconazole CL estimated for a given child to that of the representative adult. Fluconazole CL for a child was estimated by multiplying fluconazole CL_BW_ or CL_BSA_ at a given PMA by the corresponding mean BW or BSA. Fluconazole CL_BW_ or CL_BSA_ at a given PMA was estimated using the linear regression equation for the corresponding CL as a function of PMA. Heights and body weights of infants at PMA older than 40 weeks (full-term neonates) were substituted by the corresponding 50 percentile values obtained from the WHO growth chart [7]. Heights and body weights of infants at PMA younger than 40 weeks (premature neonates) were estimated according to Ahn’s table [9] containing mean values for the respective PMA.

We estimated pediatric doses of fluconazole at PMA 28–60 weeks using two empirical formulas [4, 5] as follows:

5 $$ {\mathrm{Augsberger}}^{\hbox{'}}\mathrm{s}\kern0.5em \mathrm{formula}:\kern0.5em Dose=\frac{weight(kg)\times 1.5+10}{100}\times adult\ dose $$

6 $$ {\mathrm{Crawford}}^{\hbox{'}}\mathrm{s}\kern0.5em \mathrm{formula}:\kern0.5em Dose=\frac{BSA\ \left({m}^2\right)}{1.73}\times adult\ dose $$

We also calculated the recommended doses of the drug according to the descriptions in the current prescribing information [3]. Specifically, daily doses of 3 mg/kg and 3 to 6 mg/kg are recommended for the treatment of systemic candidiasis and cryptococcosis, respectively, for infants and children older than 1 month. However, neonates younger than 2 weeks and those aged 3–4 weeks are recommended to receive the same daily doses of the drug as in older children, but every 72 and 48 h, respectively. The current prescribing information for fluconazole does not contain dose recommendation for premature neonates [3]. We calculated the daily doses of fluconazole according to these recommendations for neonates and infants younger than PMA 60 weeks.

### Statistical analysis

All statistical analyses were conducted using JMP Pro (ver. 12.2.0, SAS Institute Inc. USA). Correlation between fluconazole CL and PMA in neonates and infants, and correlation between drug CL and chronological age in children and adults were analyzed by the least-squares linear regression. Non-linear least square regression method was used to analyze the sigmoidal hyperbolic model between GFR and PMA for neonates and infants. A *p* value less than 0.05 was considered significant.

## Results

### Developmental changes of pharmacokinetic parameters of fluconazole in pediatric and adult patients

Forty-two pharmacokinetic datasets of 55 neonates or infants at PMA 27–58 weeks were obtained from the literature [14–22] (Table 1). While the information about the countries where the studies were conducted is available, no information was given in literature regarding the ethnicity of the patients. In some studies [14, 18, 20], only means or ranges for the group were available. Saxen et al. [14] conducted pharmacokinetic studies in 7 patients: 3 times for 4 patients and twice for 3 patients at 6 mg/kg. Nahata et al. [21], conducted pharmacokinetic studies in 2 patients by intravenous and oral administrations 1 week apart and in 4 patients only by an oral administration. Wenzl et al. [22] studied pharmacokinetics of the drug in one patient twice with different doses. Collectively, the doses of fluconazole studied were 3–6 mg/kg for oral administration and 2–25 mg/kg for intravenous administration. There were large inter-individual differences in elimination half-life (t_1/2_) and CL: t_1/2_ ranged from 10.7 to 88.6 h and CL normalized to body weight (CL_BW_) ranged from 0.16 to 1.18 mL/min/kg.

**Table 1:** Pharmacokinetic parameters of fluconazole in neonates and infants

Reference	N	Country	PMA (weeks)	Dose (mg/kg)	Route	AUC_(0-∞)_ (μg・h/mL)	t_1/2_ (h)	Vd (L/kg)	CL_BW_ (mL/min/kg)
Saxen et al. [14]	7	Finland	27 28 29^a^	6	IV	NA	55.2-88.6	1.18–2.25	0.18–0.52
Krzeska et al. [15]	14	Poland	41–58	3	IV	42.3–156.0	10.7–41.8	0.76–2.60	0.32–1.18
Kondo et al. [16]	4	Japan	27–33	2	IV	NA	46.2–49.4	1.07–1.35	0.25–0.33
Seki et al. [17]	6	Japan	29–42	3	IV	56.7–90.9	31.6–52.6	0.57–1.01	NA
Piper et al. [18]	8	USA	39^b^	25 (loading) 12 (maintenance)	IV	479.0^b^ 347-496^c^	56^b^ 26-80^c^	1.05^b^ 0.86–1.46^c^	0.27^b^ 0.22–0.35^c^
Wiest et al. [19]	1	USA	32	6	IV	NA	37.4	1.2	0.33
Fujii et al. [20]	6 1	Japan	40–44	3	IV PO	72.1^b^ 54.0	37.4^b^ 41.2	0.81^b^ 0.99	NA
Nahata et al. [21]	2^d^ 6	USA	30–43	6	IV PO	340.8, 425.3 340.5–636.1	NA	NA	0.16–0.29
Wenzl et al. [22]	2	Germany	36–56	4–6	PO	162.0–233.0	27.0–45.0	1.21–1.88	NA

*N* number of patients, *PMA* post-menstrual age, *NA* not available, *AUC* area under the curve, *t*_*1/2*_ half-life, *Vd* volume of distribution, *CL*_*BW*_ clearance normalized to body weight

In the study of Saxen et al. [14], data were given as ranges of means for patients who received fluconazole at different PMA. In the studies of Fujii et al. [20] and Piper et al. [18], data were given as mean for individual groups. In the studies of Krzeska et al. [15], Kondo et al. [16], Seki et al. [17], Nahata et al. [21] and Wenzl et al. [22], data were given as ranges for individual patients’ values

^a^*n* = 4, ^b^mean, ^c^interquartile range, ^d^the same patients were also studied in PO route

Sixteen pharmacokinetic datasets were retrieved from 60 children aged from 0.3 to 18.3 years (Table 2) [20, 23–26]. Specifically, Fujii et al. [20] reported the results of a multicenter clinical trial of fluconazole in which the drug was administered to 72 children and antimicrobial effects were studied. In their report, the pharmacokinetics of the drug intravenously or orally administered at different doses (3, 6, and 12 mg/kg) was studied in 7 neonates and 17 children, but only mean pharmacokinetic parameters for patients who received the same dose via the same route were available. In the study of Seay et al. [25], each child received the drug by intravenous and oral administrations and the data obtained from both routes were fitted to a pharmacokinetic model simultaneously. No data were available from children aged from 2 to 4 years. There was large inter-individual variability in the PK data: t_1/2_ ranged from 11.9 to 42.3 h.

**Table 2:** Mean or individual pharmacokinetic parameters of fluconazole in children and adults

		Reference	N	Country	Age (years)	Dose (mg)	Route	AUC (μg・h/mL)	t_1/2_ (h)	Vd (L/kg)	CL (mL/min)	CL (mL/min/kg)
Children		Sato et al. [22]	1	Japan	6	3^a^	IV	91.5	11.9	0.42	NA	NA
		Lee et al. [24]	24	USA	5–15	2-8^a^	IV	76-201	17.4	0.86	21.4	NA
		Seay et al. [25]	10	USA	1.8–15.9	3-6^a^	IV/PO	NA	15.6	0.77	16.8	0.63
		Fujii et al. [20]	17	Japan	0.3–18.3	3-12^a^	IV/PO	95.0-200.9	17.3–23.5	0.49–0.69	NA	NA
		Nahata et al. [26]	8	USA	6–13	2-8^a^	PO	84.9-684.3	19.8–42.3	NA	NA	NA
Adults	Healthy volunteers	Shiba et al. [27]	8	Japan	20–22	25–100	IV/PO	19.2–86.9	28.6–33.4	41.3–59.4^b^	NA	NA
		Ripa et al. [28]	18	Italy	20–37	50–150	IV/PO	39.8–114.2	29.7–32.2	50.9–53.4^b^	20.6–21.0	NA
		Takebe et al. [29]	10	Japan	23–34	200	PO	253.1	44.7	49.9^b^	NA	NA
		Humphrey et al. [30]	4	UK	18–45	1^a^	PO	NA	22	0.7	NA	0.40
		Thorpe et al. [31]	14	UK	21–29	100	PO	93.0	NA	NA	NA	NA
		Toon et al. [32]	5	UK	22–66	50	PO	NA	31.2	0.91	23.8	NA
		Jovanovic et al. [33]	24	Serbia	22–48	150	PO	107.0	35.1	NA	NA	NA
		Yeates et al. [34]	10	Germany	27	100	IV	NA	35.0	0.94	23.0	NA
		Tett et al. [35]	10	Australia	18–45	50–400	IV	NA	31–46	42-78^b^	14.8–26.2	NA
	AIDS Patients	Yeates et al. [34]	10	Germany	35	100	IV	NA	37.0	0.84	17.0	NA
		Tett et al. [35]	11	Australia	25–50	50–400	IV	NA	25–69	31-56^b^	8.8–19.8	NA
		DeMuria et al. [36]	10	USA	29–38	100	IV	67.2–188	21.8–75.2	35.8–59.9^b^	8.9–24.8	NA
		Chin et al. [37]	1	Canada	37	400	IV	NA	34.2	28.1^b^	9.5	0.19
	Vaginal candidiasis	Houang et al. [38]	9	UK	23–41	150	PO	NA	30.2	0.84	NA	0.32
	Burn patients	Boucher et al. [39]	8	USA	24–65	400	IV	152–276	14.4–38.4	0.48–0.96	NA	0.19–0.53

*N* number of patients, *NA* not available, *AUC* area under the curve, *t*_*1/2*_ half-life, *Vd* volume of distribution, *CL*_*BW*_ clearance normalized to body weight

In the studies of Lee et al. [24], Fujii et al. [20], Shiba et al. [27], Ripa et al. [28], data were given as ranges of means for patients who received fluconazole at different doses or routes. In the studies of Seay et al. [25], Takebe et al. [29], Humphrey et al. [30], Thorpe et al. [31], Toon et al. [32], Jonanovic et al. [33], Yeates et al. [34] and Houang et al. [38], data were given as mean values for the individual groups. In the studies of Nahata et al. [26], Tett et al. [35], DeMuria et al. [36] and Boucher et al. [39], data were given as ranges for individual patients’ values

^a^mg/kg, ^b^L

For adults, 60 pharmacokinetic datasets were retrieved from those aged from 18 to 65 years (103 healthy volunteers, 32 patients with AIDS, 9 patients with vaginal candidiasis, and 8 patients with burn) [27–39]. In the reports of Shiba et al. [27] and DeMuria et al. [36], all patients received the drug by intravenous and oral routes on different occasions, For these patients, only the pharmacokinetic data obtained after an intravenous administration were included in analysis. In the report of Ripa et al. [28], 18 patients were allocated to 3 groups (*n* = 6 each) and received the drug at different doses: 50 or 150 mg orally, or 100 mg intravenously. We calculated fluconazole CL from plasma concentration–time data obtained after oral administration assuming that the oral bioavailability of fluconazole was 94% [6]. Doses employed for oral administration were from 25 to 200 mg and those for intravenous administration were from 25 to 400 mg. T_1/2_ ranged from 14.4 to 75.2 h; CL ranged from 8.8 to 26.2 mL/min; and CL_BW_ ranged from 0.19 to 0.63 mL/min/kg (Table 2).

### Relationship between fluconazole CL and PMA in neonates and infants compared to that in children and adults

**Fig. 1:**
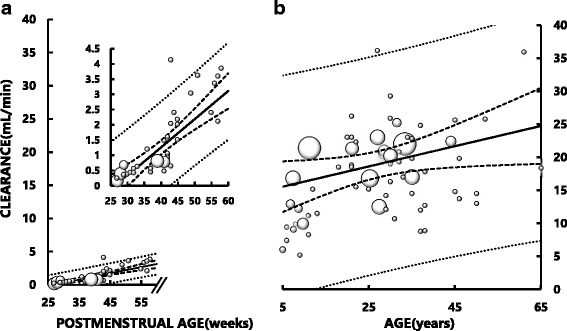
Relationship between systemic fluconazole clearance (CL) and postmenstrual age (PMA) in neonates and infants (**a**) and between fluconazole CL and age in children aged older than 5 years and adults (**b**). In the inset of A, the data in infants are plotted on a magnified ordinate scale for better visibility. The area of each symbol (○) is in proportion to the number of dataset. There is a significant correlation between fluconazole CL and PMA in infants (*Y* = 0.093X - 2.45, *r* = 0.81, *p* < 0.001), and between fluconazole CL and age in young children and adults (*Y* = 0.154X + 14.74, *r* = 0.36, *p* < 0.005). Solid lines represent the least square regression lines. Bold dotted lines represent the upper and lower 95% confidence intervals of the regression lines. Fine dotted lines represent the 95% confidence intervals of the datasets. Note that the scales of the abscissae are different in (**a**) and (**b**). No data were available for children aged between PMA 60 weeks and 5 years

While fluconazole CL in neonates and infants was much lower than that in children older than 5 years and adults due mainly to smaller body size, there was a significant positive correlation between fluconazole CL and PMA in neonates and infants (Fig. 1a): *Y* = 0.093X – 2.45, *r* = 0.81 (*p* < 0.001). A significant correlation between fluconazole CL and age was also observed in young children and adults (Fig. 1b): *Y* = 0.154X + 14.74, *r* = 0.36 (*p* < 0.005).

**Fig. 2:**
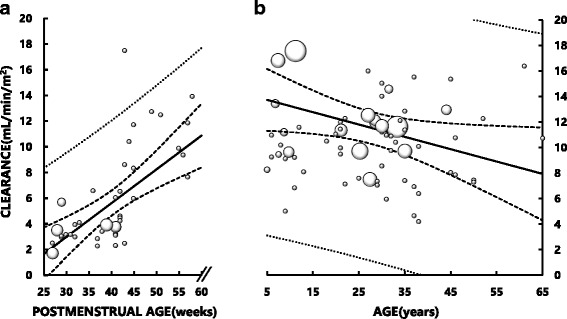
Relationship between fluconazole clearance normalized to body surface area (BSA) (CL_BSA_) and postmenstrual age (PMA) in neonates and infants (**a**) and between fluconazole CL_BSA_ and age in children older than 5 years and adults (**b**). The area of each symbol (○) is in proportion to the number of dataset. BSA of premature infants and neonates was estimated by Haycock’s formula, and that of children and adults by Dubois’s formula. There is a significant correlation between fluconazole CL_BSA_ and PMA in neonates and infants (*Y* = 0.263X -4.92, *r* = 0.68, *p* < 0.001), and between fluconazole CL_BSA_ and age in young children and adults (*Y* = − 0.097X + 14.22, *r* = − 0.36, *p* < 0.005). Solid lines represent the least square regression lines. Bold dotted lines represent the upper and lower 95% confidence intervals of the regression lines. Fine dotted lines represent the 95% confidence intervals of all datasets

To evaluate the development of fluconazole CL in size-adjusted values in subjects ranging from neonates to adults having different body sizes, we normalized fluconazole CL to BSA (CL_BSA_) and body weight (CL_BW_). CL_BSA_ in neonates was 1/3 to 1/4 of that in adults, but it increased rapidly after birth and reached largely the adult values at PMA 60 weeks (approximately 5 months postpartum) (Fig. 2a). There was a significant positive correlation between CL_BSA_ and PMA in neonates and infants: CL_BSA_: *Y* = 0.263X – 4.92, *r* = 0.68 (*p* < 0.001). In contrast, a significant negative correlation between CL_BSA_ and chronological age was found in children older than 5 years and adults (Fig. 2b): *Y* = − 0.097X + 14.22, *r* = − 0.36 (*p* < 0.005).

**Fig. 3:**
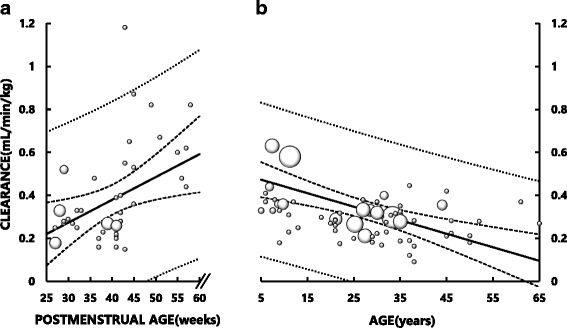
Relationships between fluconazole clearance normalized to body weight (BW) (CL_BW_) and postmenstrual age (PMA) in neonates and infants (**a**) and between fluconazole CL_BW_ and age in children older than 5 years and adults (**b**). The area of each symbol (○) is in proportion to the number of datasets. There is a significant correlation between fluconazole CL_BW_ and PMA in neonates and infants (*Y* = 0.011X – 0.04, *r* = 0.46, *p* < 0.005), and between fluconazole CL_BW_ and age in young children and adults (*Y* = − 0.006X + 0.50, *r* = − 0.60, *p* < 0.001). Solid lines represent the least square regression lines. Bold dotted lines represent the upper and lower 95% confidence intervals of the regression lines. Fine dotted lines represent the 95% confidence intervals of all datasets

Fluconazole CL_BW_ in neonates was comparable to that in adults (Fig. 3a). There was also a significant correlation between CL_BW_ and PMA (*Y* = 0.011X – 0.04, *r* = 0.46, *p* < 0.005), and CL_BW_ at PMA 60 weeks apparently increased to a level exceeding the adult values (Fig. 3a). In contrast, there was a significant negative correlation between CL_BW_ and age in children older than 5 years and adults (Fig. 3b): *Y* = − 0.006X + 0.50, *r* = − 0.60 (*p* < 0.001).

### Ontogeny of GFR in neonates to young children

A total of 187 relevant datasets of GFR and age in children, spanning premature neonates through children aged 12 years, were retrieved from the articles of Rubin et al. [40], Fawer et al. [41], Coulthard [42] and van der Heijden et al. [43]. Briefly, Rubin et al. [40] reported individual data of GFR and age for 63 pediatric patients with chronological ages ranging from 2 days to 12 years and body weights ranging from 2.4 to 35.5 kg. They measured GFR by mannitol clearance. We assumed that no premature neonates were studied in their study, since there were no descriptions in the article suggesting premature labor and all neonates had gestational ages of 40 weeks or longer. Fawer et al. [41] studied GFR in 44 pediatric patients having gestational ages ranging from 28 to 43 weeks (postpartum ages from 0.5 to 19 days) and body weights at birth ranging from 1.1 to 5.38 kg. They measured GFR by inulin clearance. Coulthard [42] studied GFR in 39 pediatric patients with gestational ages ranging from 27 to 40 weeks (chronological ages from 2 to 33 days) and body weights ranging from 0.85 to 3.85 kg. They measured GFR by inulin clearance. Van der Heijden et al. [43] studied GFR in 41 pediatric patients with gestational ages ranging from 27 to 36 weeks (chronological ages from 3 to 11 days) and body weights at birth ranging from 0.81 to 2.74 kg. They measured GFR by inulin clearance. Individual body weights and heights of full-term infants were available in the article of Rubin et al. [40], whereas only individual body heights were available in the other articles [41–43]. Therefore, we decided to estimate BSA for these pediatric subjects using Ahn’s equation (Eq. 3).

**Fig. 4:**
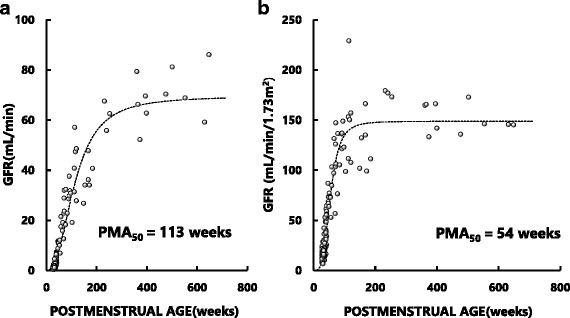
Developmental time courses of unadjusted glomerular filtration rate (GFR) (**a**) and GFR normalized to body surface area (BSA) of 1.73m^2^ (GFR_BSA_) (**b**) as a function of PMA in neonates, infants, and children. The original datasets were retrieved from the reports of Rubin et al. (40), Coulthard et al. (42), Fawer et al. (41) and van der Heijden et al. (43). Details of the sigmoidal hyperbolic model are described in the text

Developmental changes of GFR and GFR_BSA_ analyzed as a function of PMA are shown in Fig. 4a and b, respectively. By fitting the retrieved datasets to a sigmoidal hyperbolic function model, we obtained the following parameters. For the GFR–PMA relationship, GFR_max_ was 70 mL/min; PMA_50_ was 113 weeks; and Hill coefficient was 2.5 (Fig. 4a). For the GFR_BSA_–PMA relationship, GFR_BSAmax_ was 149 mL/min/1.73m^2^; PMA_50_ was 54 weeks; and Hill coefficient was 3.7 (Fig. 4b).

### Relationship between fluconazole CL and GFR in neonates and infants

**Fig. 5:**
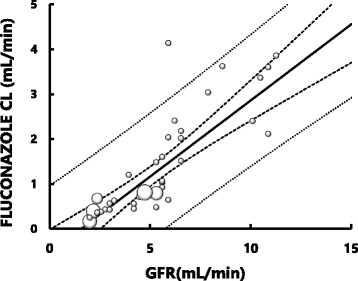
Relationship between fluconazole clearance (CL) (mL/min) and glomerular filtration rate (GFR) (mL/min) in neonates and infants younger than PMA 60 weeks. The area of each symbol (○) is in proportion to the number of datasets. Bold solid line shows the regression line of the data. Bold dotted lines show the upper and lower limits of 95% confidence intervals of the regression line. Fine dotted lines show the upper and lower 95% confidence intervals for the datasets. A significant correlation is observed: *Y* = 0.34X - 0.53, *r* = 0.84 (*p* < 0.001)

A significant correlation was observed between fluconazole CL and GFR in neonates and infants: *Y* = 0.34X – 0.53, *r* = 0.84, *p* < 0.001 (Fig. 5). Since we have shown that GFR in a neonate or infant at a given PMA can be estimated using the sigmoidal hyperbolic equation (Fig. 4), fluconazole CL for the corresponding neonate or infant may be estimated by substituting his or her GFR into the regression equation shown in Fig. 5.

### Comparisons between fluconazole doses for neonates and infants predicted using empirical formulas and using CL-based formulas in reference to recommended doses in prescribing information

**Fig. 6:**
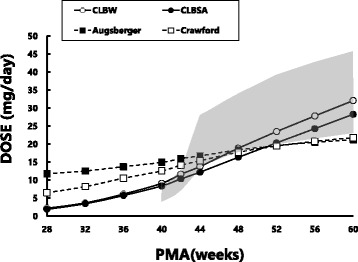
Comparisons of fluconazole doses for neonates and infants estimated using various formulas for children at PMA ranging from 28 to 60 weeks. Open (○) and closed circles (●) connected with solid lines represent doses predicted by fluconazole clearance normalized to body weight (CL_BW_) and fluconazole clearance normalized to body surface area (CL_BSA_), respectively in the present study. Open (□) and closed squares (■) represent those predicted by the Crawford’s and Augsberger’s formulas, respectively. The shaded area represents doses calculated according to the recommended doses for treating systemic candidiasis for children (3 to 6 mg/kg/day) in the latest prescribing information [3]

Compared to the daily doses of fluconazole recommended in the prescribing information for neonates and infants [3], Augsberger’s and Crawford’s formulas appeared to overestimate the doses for neonates at PMA 40–42 weeks, and appeared to underestimate the doses for infants at PMA 52 weeks and older (Fig. 6). In contrast, daily doses of fluconazole for neonates and infants predicted by our CL-based method were essentially within the doses recommended by the prescribing information for infants at PMA 40–60 weeks. For premature neonates, the doses predicted by our method was approximately 50% smaller than those predicted by the 2 empirical formulas.

## Discussion

To the best of our knowledge, the present study is the first to demonstrate significant increases in size-unadjusted fluconazole CL and size-adjusted fluconazole CL (CL_BSA_ and CL_BW_) as a function of PMA in neonates and infants (Figs. 1, 2 and 3). Particularly, CL_BSA_ in neonates was substantially lower than adult value but increased rapidly after birth reaching the level of adults at approximately PMA 60 weeks (Fig. 2a). These data suggest that the development of fluconazole CL may be attributed not only to an increase in size of body (and organs associated with the elimination of fluconazole) but also to functional maturation of the organ(s) which is associated with elimination of fluconazole. Since fluconazole is eliminated mainly by the kidneys, our data imply rapid and substantial maturation in nephron function in these pediatric subjects. Our notion may be supported by the findings that GFR_BSA_ also increased rapidly after birth, reaching a plateau that is largely comparable to adult levels by PMA 100 weeks (approximately age 1.5 years) (Fig. 4). Our data thus indicate that doses of fluconazole for children younger than 2 years should not be estimated simply from usual adult doses using BSA or BW as size-scaling factors, but should be estimated taking into account the development of fluconazole CL in association with functional maturation of nephrons (i.e., GFR_BSA_).

We found that CL_BSA_ obtained from neonates at PMA younger than 40 weeks was approximately 1/4 to 1/3 of adult values (Fig. 2a), whereas the corresponding CL_BW_ appeared largely comparable to adult values (Fig. 3a). A previous study has shown that the developmental time courses of organ size or mass (such as kidneys) and BSA are almost identical [2]. In this context, we surmise that neonates may have substantially immature fluconazole CL per unit kidney mass (i.e., CL normalized to BSA), compared to adults (Fig. 2a and b). Similar findings have been reported for other drugs [44–46]. These findings may be explained by a difference in the developmental time course of body weight and that of organs. The development of body weight has been shown to lag behind that of organ size and BSA [2]. As a result, the ratios of organ mass (such as liver and kidney mass) per body weight in children are greater, maximally two-fold, than those in adults. Consequently, fluconazole CL_BW_ in neonates might have been overestimated and thereby appeared similar to that in adults, and fluconazole CL_BW_ in infants around PMA 60 weeks and children aged 5 to 10 years might have been overestimated and appear to be greater than that in adults (Fig. 3a and b).

We found that while unadjusted GFR (mL/min) in neonates were substantially lower than those in adults, GFR increased rapidly as shown by a PMA_50_ of 113 weeks and appeared to reach a plateau around PMA 600 weeks or age 10 years (Fig. 4a). Rodin et al. [12] reported data being similar to ours and analyzed the curvilinear data with the sigmoidal model. We also adopted the model in the present study. Interestingly, the plateau level (149 mL/min/1.73m^2^) for children was comparable to that of a representative adult male (130 mL/min/1.73m^2^) [47] (Fig. 4b). Since GFR_BSA_ represents the nephron function per standardized mass of kidney, this finding indicates that neonatal nephron function is very immature at birth but develops rapidly in early childhood reaching almost adult level by around age 2 years. Our findings are supported by previous studies on the ontogeny of renal morphology and physiology. A study has shown that although nephrogenesis is complete by 36 weeks of gestation, neonatal GFR is only approximately 5% of the adult value [48]. Neonatal nephron function is immature due mainly to vasoconstriction of the renal microvasculature, whereas the postnatal increase in renal mass is due almost entirely to tubular growth [48].

Assuming that plasma fluconazole concentrations required for the treatment of systemic fungal infection are comparable between children and adults, and that the typical adult dose of the drug is 100 mg/day, it is possible to estimate individual fluconazole doses for neonates or infants from adult doses using fluconazole CL of children as a scaling factor. Since there is good relationships between GFR of neonates and infants and PMA (Fig. 4) and between fluconazole CL and GFR (Fig. 5), fluconazole CM for a neonates and infants may be estimated by PMA. The fluconazole doses for neonates at PMA 40–44 weeks predicted by the two empirical dosing formulas are higher than those estimated using CL_BSA_ or CL_BW_ and those recommended in the prescribing information. In contrast, the doses estimated using CL_BSA_ or CL_BW_ were similar to those recommended in the prescribing information for systemic candidiasis (3 to 6 mg/kg/day). For infants at PMA 44 weeks and older, fluconazole doses predicted by our CL-based methods and the 2 empirical formulas are essentially within the range of doses recommended by the prescribing information.

The present study has some limitations. Since pharmacokinetic studies for children are difficult to conduct, only sparse data and datasets with incomplete clinical information are available from the literature. As a result, we often had to utilize average demographic data from subjects with comparable PMA or mean pharmacokinetic parameters for a group in estimating parameters for individual subjects. In addition, since no pharmacokinetic data were available for children from PMA 60 weeks to age 5 years, we cannot estimate the development of fluconazole CL during this period. Finally, CL-based prediction of fluconazole doses for pediatric patients with fungal infection would only be valid if therapeutic plasma drug concentrations are similar between children and adults. Since neonates and infants are immunologically less competent than older children, whether they need higher fluconazole concentrations than adults for treatment remains to be studied. The reason why CL_BW_ and CL_BSA_ showed weak, but significant, negative correlations with age (Figs. 2b and 3b) are most likely associated with an age-dependent decrease in GFR [49].

## Conclusions

This study reveals that size-normalized fluconazole CL in neonates and infants is substantially lower than that in adults, but fluconazole CL develops rapidly during early childhood. Immature nephron function in neonates and infants may contribute to immature renal clearance for fluconazole. Fluconazole doses for neonates and infants may be estimated by taking into consideration developmental change of CL_BSA_ that is associated with the development of GFR.

## Abbreviations

BSA: Body surface area
CL: Systemic clearance
CL_BSA_: Clearance normalized to BSA
CL_BW_: Clearance normalized to body weight
GFR: Glomerular filtration rate
GFR_BSA_: Glomerular filtration ratio normalized to BSA
GFR_BSA,max_: The maximum value for GFR
PMA: Postmenopausal age
PMA_50_: Postmenopausal age associated with 50% of the maximum GFR
